# Evolution and the complexity of bacteriophages

**DOI:** 10.1186/1743-422X-4-30

**Published:** 2007-03-13

**Authors:** Philip Serwer

**Affiliations:** 1 Department of Biochemistry, The University of Texas Health Science Center at San Antonio, 7703 Floyd Curl Drive, San Antonio, Texas 78229-3900, USA

## Abstract

**Background:**

The genomes of both long-genome (> 200 Kb) bacteriophages and long-genome eukaryotic viruses have cellular gene homologs whose selective advantage is not explained. These homologs add genomic and possibly biochemical complexity. Understanding their significance requires a definition of complexity that is more biochemically oriented than past empirically based definitions.

**Hypothesis:**

Initially, I propose two biochemistry-oriented definitions of complexity: either decreased randomness or increased encoded information that does not serve immediate needs. Then, I make the assumption that these two definitions are equivalent. This assumption and recent data lead to the following four-part hypothesis that explains the presence of cellular gene homologs in long bacteriophage genomes and also provides a pathway for complexity increases in prokaryotic cells: (1) Prokaryotes underwent evolutionary increases in biochemical complexity after the eukaryote/prokaryote splits. (2) Some of the complexity increases occurred via multi-step, weak selection that was both protected from strong selection and accelerated by embedding evolving cellular genes in the genomes of bacteriophages and, presumably, also archaeal viruses (first tier selection). (3) The mechanisms for retaining cellular genes in viral genomes evolved under additional, longer-term selection that was stronger (second tier selection). (4) The second tier selection was based on increased access by prokaryotic cells to improved biochemical systems. This access was achieved when DNA transfer moved to prokaryotic cells both the more evolved genes and their more competitive and complex biochemical systems.

**Testing the hypothesis:**

I propose testing this hypothesis by controlled evolution in microbial communities to (1) determine the effects of deleting individual cellular gene homologs on the growth and evolution of long genome bacteriophages and hosts, (2) find the environmental conditions that select for the presence of cellular gene homologs, (3) determine which, if any, bacteriophage genes were selected for maintaining the homologs and (4) determine the dynamics of homolog evolution.

**Implications of the hypothesis:**

This hypothesis is an explanation of evolutionary leaps in general. If accurate, it will assist both understanding and influencing the evolution of microbes and their communities. Analysis of evolutionary complexity increase for at least prokaryotes should include analysis of genomes of long-genome bacteriophages.

## 1. Background

### Empirical studies of the genomes of viruses

Bacteriophages and eukaryotic viruses with comparatively long double-stranded DNA genomes have genes homologous to cellular genes. For illustrating the surprising character of this observation, the shorter viral genomes serve as a baseline. Specifically, the shorter-genome, virulent double-stranded DNA bacteriophages, such as φ29 (genome length = 19.3 Kb [1]), T3 (genome length = 38.2 Kb [2]) and T7 (genome length = 39.9 Kb [2]), have genes most of which are tightly packed. The φ29, T3 and T7 genes with identified functions almost always have a role in bacteriophage-specific biochemistry [1-3].

The virulent bacteriophage T4 has a longer, 168 Kb genome. The greater length of the T4 genome is explained, in part, by the more numerous components of T4 structure, especially the tail. However, not so easily explained is the informatics-detected presence in the T4 genome of homologs of transfer RNA genes, genes for nucleotide metabolism, DNA repair enzymes [4] and, in the case of a T4-related bacteriophage, genes for an NAD salvage pathway [5]; all informatics discussed here uses the genomic base sequence as input. None of the T4 cellular gene homologs are present in the shorter genomes of φ29, T3 and T7 [1-3].

In an extension of this pattern, a larger collection of cellular gene homologs is informatics-detected in the even longer 280 Kb genome of bacteriophage φKZ [6]. Remarkable is the presence of φKZ genes that (a) encode enzymes with a wide range of metabolic functions and (b) have closest homologs that are from bacteria that are not φKZ hosts and that sometimes are distantly related to the φKZ host, *Pseudomonas aeruginosa*. These latter genes encode several RNA polymerases, DNA repair proteins, cell division proteins and stringent starvation protein [6,7]. The presence of these genes is not well explained by direct need for the gene products in the process of bacteriophage reproduction, though the gene products can assist virus propagation by assisting the host.

The most frequently sequenced long viral genomes are from viruses with eukaryotic hosts. Again, the long eukaryotic viral genomes have cellular gene homologs whose presence in a viral genome is unexplained. For example, giant (313 – 415 Kb genome) phycodnavirus virus genomes have informatics-detected, cellular gene homologs that include genes for tRNAs, ubiquitin, UV-specific DNA repair enzyme, transcriptional elongation factor TFIIS, chitin synthase, RNA polymerase subunits, N-acetylglucosaminyl transferase and multiple enzymes in each of several metabolic pathways, including those for synthesis of hyaluranan, sphingolipid, fucose, and polyamines [8-10].

The longest viral genome is the 1,200 Kb genome of the phycodnavirus-related mimivirus of *Acanthamoeba polyphaga*. Mimivirus also has the largest collection of cellular gene homologs. Informatics-detected mimivirus genes include homologs for 40 bacterial proteins and 46 eukaryotic cell proteins. The mimivirus genes include genes for 4 aminoacyl tRNA synthetases, 33 enzymes of carbohydrate metabolism, 3 signaling receptors and several translation factors among many other genes whose products might assist virus propagation by assisting the host, but are not expected to have virus-specific functions [9,11-14]. Conservation of a putative promoter sequence indicates that the gene products are made and functional [15]. The existence of these genes in viral genomes is currently considered a major mystery because they increase the length of viral genomes without producing any known selective advantage [12].

### Theoretical framework

A selective advantage must exist for the cellular gene homologs of long-genome viruses. To establish a theoretical framework for determining what this selective advantage is, I make a first assumption that the cellular gene homologs of long-genome viruses introduce increased complexity that is related, in some way, to increased complexity at the level of biochemistry. Next, I will use past experimental work to obtain a definition of complexity that is applicable to biochemistry. This process led to a departure from past thinking because, in the past, empirically based definitions of biological complexity have focused on those properties of higher eukaryotes that can be quantified either via length and randomness of genome sequence [16] or via simple characteristics of structure [17-21]. These latter definitions are not meant to be fundamental to complexity at the level of biochemistry.

In search of a fundamental definition of change in (not absolute) biochemistry-based complexity, two well-investigated examples are considered here. Both examples involve the transfer of genes to bacteria by bacteriophage vectors. The first example is bacterial gene transfer via bacteriophage-based generalized transduction. Generalized transduction happens randomly with regard to the genes transferred [22-24].

The second example is bacterial gene transfer via bacteriophage-based lysogenic conversion. In contrast to generalized transduction, lysogenic conversion happens with specificity for a specific gene that, based on past selections, will promote future invasion of a host by the converted bacterial cell. The basis of the specificity includes encoded, evolutionary selection-derived memory of the usefulness of the gene product [25,26]. This encoded memory-based specificity sometimes occurs by making the gene product part of the bacteriophage particle. Examples include hyaluronidase [27], as well as adhesion proteins for bacterial host attachment [25]. Thus, the encoded memory-based specificity is biochemically complex in that it comes from not only the product of the gene transferred, but also from other, interacting gene products. Note that information about the future is derived from selection in past circumstances that mimic future circumstances. No other source of information is involved.

From the above example, lysogenic conversion is more complex than generalized transduction by two definitions of increased complexity: **(a) decreased randomness that does not serve immediate needs and (b) increased encoded information that does not serve immediate needs**. Though these two definitions are not necessarily completely equivalent, the second assumption made here is that the above two definitions of change in complexity are completely equivalent in content (equivalence assumption). The second of these two definitions partially overlaps the following previous definition proposed in the context of the evolution of "digital organisms" [28]: encoded "information about the environment that can be used to make predictions about it".

Blood clotting provides an empirical application and test of the equivalence assumption in the case of eukaryotes. Blood clotting is complex by the second definition, based on the multiple factors and the cascade needed to initiate clotting. Blood clotting is also an event in which randomness (that will cause clotting either too rapid or too slow) is minimized [29,30]. Randomness in blood clotting is a major selective disadvantage for survival.

### Late-evolving complexity of bacteriophage biochemistry

**Figure 1:**
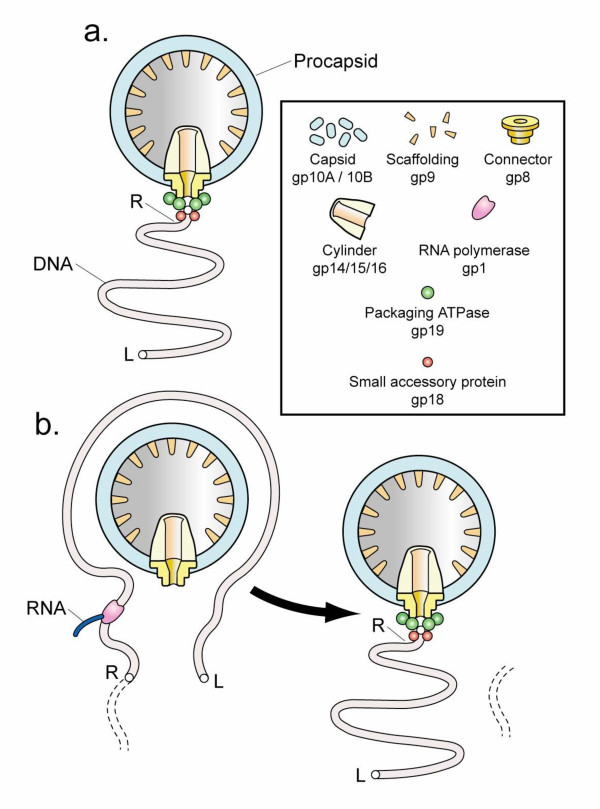
Initiation of DNA packaging by the closely related bacteriophages, T3 and T7. (a) Initiation is illustrated for the simplest DNA packaging. This packaging has a monomeric DNA substrate and was demonstrated for T3 and assumed for T7. Packaging of this type occurs only *in vitro*, as far as is known (review [31,32]). (b) Initiation is illustrated for the more complex DNA packaging that occurs *in vivo *for both T3 and T7 (review [31,32]). In (b), the DNA substrate is an end-to-end joined concatemeric DNA for which only one monomer is completely shown. Dashed lines in (b) indicate part of another monomer within the concatemer. The following details of the concatemer are omitted for simplicity: replication forks and interaction among different procapsids (review [32]). The various proteins and protein assemblies of the initiation complex, including the connector and DNA packaging ATPase, are identified in the rectangular box. Proteins have both descriptive names and names based on gene number [2], preceded by gp. The letter, R, indicates the right end of the mature DNA molecule; the letter, L, indicates the left end.

Although the smaller bacteriophage genomes lack cellular gene homologs, some aspects of small bacteriophage multiplication have undergone recognizable increase in biochemical complexity by the second definition of the previous section. One such aspect is DNA packaging. All known double-stranded DNA bacteriophages produce progeny by, first, assembling a DNA-free capsid (procapsid) and, then, binding and packaging the DNA genome. Figure 1a shows the initiation complex for packaging bacteriophage T3 DNA in a simplified *in vitro *system. In Figure 1a, the DNA molecule binds a DNA-binding accessory protein (also called gp18) that binds a DNA packaging ATPase, also called gp19. The DNA packaging ATPase binds a 12-fold symmetric ring (connector) with an axial hole. The DNA molecule is subsequently packaged through this hole into a cavity of an outer protein shell (capsid) (reviews [1,31,32]). The structure of the capsid changes during DNA packaging (not shown in Figure 1).

Even though T3 *in vitro *DNA packaging is efficient with the initiation complex of Figure 1a[31], the initiation complex used *in vivo *by both T3 and its close relative, T7, has more complexity. The additional complexity comes from packaging initiation *in vivo *that depends on transcription by a bacteriophage-encoded RNA polymerase (also called gp1 [33-35]) (illustrated in Figure 1b). At least three proteins (gp1, gp18, gp19) have encoded information for this interaction. Based on the equivalence assumption, the additional complexity at the initiation of packaging (second definition of complexity) should provide decrease in the randomness of an event of the subsequent process of DNA packaging (first definition of complexity).

In this case, the literature already supports the equivalence assumption by describing two possibilities for what this event is (both possibilities can be correct): (a) The first possibility is entry of the DNA molecule into the cavity of the capsid. The selective advantage is controlled (less random) initiation of entry so that entry events are not so numerous that ATP is consumed to the point that no genome completes packaging [32]. Evidence also exists for complexity of this type at the level of the T7 DNA packaging process itself [32]. (b) The second possibility is termination of packaging, an event that includes both selective replication of a terminally repeated DNA sequence and cleavage of the genome from a longer, concatemeric DNA molecule. The selective advantage is that a genome is not cleaved from a concatemer until replication of its terminal repeat is completed [35].

Furthermore, the complexity added by RNA polymerase-dependence of the initiation of T3/T7 DNA packaging was a product of comparatively recent evolution, based on the following two observations: (a) T3/T7 relatives exist that do not have the RNA polymerase in their genomes. These relatives are thought to be less evolved in their transcription [36,37]. (b) RNA polymerase-dependence of the initiation of DNA packaging has not yet been found in a eukaryotic virus, even though eukaryotic viruses have common ancestors with bacteriophages (below) and are more intensely studied than bacteriophages. Thus, the chance is high that transcription dependence of T3/T7 DNA packaging evolved after the split between bacteria and eukarya, i.e., after about 1.6 billion years ago (review [38]). The data support the same conclusion for transfer to archaea of bacterial chaperonin, *hsp*70. These data include the absence of *hsp*70 from many archaea (review [39]).

Post-split evolution of prokaryotic complexity is a phenomenon often overlooked during analysis focused on eukaryotes (see, for example ref. [21]). One reason appears to be that non-adaptive expansion of genome size is thought to be the dominant genome length-determining theme in eukaryotes [16]. This expansion is an entropic response to a low population density-induced reduction of competition. Environmental population densities are not known for most bacteriophage strains. But, the number of bacteriophages produced per cell (typically over 100 [40]) and the total environmental bacteriophage concentrations (10^8 ^– 10^9 ^per gm in soil [41,42]) indicate that this type of non-adaptive genome expansion is unlikely in the case of bacteriophages. In support, long genome bacteriophages, such as φKZ [6,7], have open reading frames highly compacted, as though under constant selection.

Neither the evolution of post-split complexity nor the presence of cellular gene homologs in the genomes of long genome viruses is currently explained with a hypothesis that can be tested. The hypothesis of the next section fills this intellectual gap. This hypothesis can be tested because of both short life cycles of bacteriophages and recent advances in isolation and sequencing bacteriophage genomes.

## 2. A hypothesis for the selective advantage of cellular gene homologs in long bacteriophage genomes

Although the above observations indicate that some post-split increase in biochemical complexity has occurred for bacteriophages, the following observations indicate that some basics evolved pre-split: structural similarities among the outer shell proteins of bacteriophages, archaeal viruses and eukaryotic viruses [43-48]. The structural similarity extends to the DNA packaging ATPases [49,50]. From these data, viral identity (also called viral self) is based on the secondary/tertiary/quaternary structure of the proteins that constitute the viral particle [44-46,51].

Thus, the data indicate that post-split viruses are independently evolving and not simply post-split breakaways from their hosts. Furthermore, the data indicate a predominantly prokaryotic gene pool worldwide with more (about 10 ×) bacteriophages than bacteria (reviews [52-55]). Thus, the bacteriophage cellular gene homologs exist in the context of viral evolution that has the potential for major impact on prokaryotic cells.

Together with the above data, the equivalence assumption is used here to derive a hypothesis to explain the selective advantage of the genomic complexity introduced by the presence of cellular gene homologs in bacteriophages (second definition of complexity). The equivalence assumption produces the conclusion that the selective advantage is reduction of randomness (first definition of complexity) of an event that both has and will occur for all of the wide-ranging host-like biochemical systems encoded by these genes. The most fundamental aspect of the hypothesis presented here is that this event is proposed to be evolution itself, i.e., evolution of biochemical systems encoded by the genes of host bacteria and possibly other bacteria that exchange DNA with the host. The following are the details of the hypothesis:

(1) Increase in the biochemical complexity of prokaryotic cells and their viruses occurred after the eukaryote/prokaryote splits (support is above).

(2) In the case of prokaryotic cells, the coding for at least some of this increase initially evolved not via genes in the cellular genome, but via host cellular gene homologs in the genomes of long-genome, rapidly evolving prokaryotic viruses. The products of the host cellular gene homologs involved were not participants in bacteriophage-specific events, but did assist bacteriophage infection by assisting the host. Thus, direct selective pressure occurred for these genes to evolve, though the genes were non-essential. The result was multi-step evolution in which intermediate steps did not necessarily provide enough selective advantage to survive life or death situations (first tier selection). However, the end products of some multi-step selections did provide this type of advantage, as discussed further in the next two paragraphs. The cellular gene homologs of today's long-genome bacteriophages are descendants of these earlier homologs. A potential (not proven) ongoing example of an infection-assisting, viral genome-encoded cellular gene homolog is the host photosynthetic gene, psbA, present in the genomes of 8 of 9 sequenced cyanophages. The host-encoded psbA gene product is subjected to rapid turnover during infection. The assumption is that expression of the bacteriophage gene compensates for the rapid turnover [56].

(3) In addition to the multi-step first tier selection undergone by the bacteriophage-associated cellular gene homologs, additional selection and evolution occurred for the genes whose products maintained cellular gene homologs within a bacteriophage genome (second tier selection). The second tier selection caused the retention and improvement of the first tier selection because of the long-term selective advantage of multi-step evolution of complex biochemical systems when transferred (ultimately) to the host. That is to say, selection for complex systems was two-tiered. The first tier was based on immediate (classical), though potentially minor, short-range selective advantage at each step. The second tier was based on long-range, major selective advantage that arose from retaining and improving the first tier.

(4) DNA exchange moved to prokaryotic hosts the biochemical systems encoded by cellular gene homologs in bacteriophage genomes. This exchange occurred repeatedly and in both directions. The host cell occasionally received a biochemical system of either (a) immediate major selective advantage or (b) major selective advantage after additional mutation. In either case, introduction or replacement of a major pathway in the host cell occurred and bacteriophage-associated host gene evolution had provided a major competitive advantage.

The advantages of bacteriophage-based host evolution were the following: (a) Each bacteriophage gene duplicated and, therefore, evolved at comparatively high rate when under selective pressure. Bacteriophages typically have (and presumably had) a burst size of over 100 infective particles produced in a time span of 0.5 – 2.0 hr [40]. (b) Bacteriophages engaged in horizontal gene transfer among different hosts within microbial communities, thereby increasing the rate of evolution via genetic exchange [54,57]. (c) Since at least some cellular gene homologs were non-essential, multi-step evolutionary "leaps" in complexity occurred even if some of a leap's component steps provided either no or only minor selective advantage. This aspect resolves a vexing problem in considering evolutionary leaps in general. Computer-simulation has shown the evolution of complex features via digital mutations that produce intermediates that are sometimes neutral or even detrimental [58].

## 3. Testing and feasibility of the prokaryotic virus complexity hypothesis

### Feasibility

The prokaryotic virus complexity hypothesis is distinguished by its second tier evolutionary selection that (a) yields bacteriophage-encoded biochemical systems that function to retain non-essential genes for the first tier and (b) does so with a time delay because of the gene transfer and possibly gene transformation events that occur before the selective advantage is realized. Although retention of non-essential genes initially might seem unlikely, bacteriophages are already known to have systems to retain genes that, while not cellular gene homologs, are non-essential for growth on laboratory host strains. Presumably, these latter genes are essential for growth on other strains and will be called conditionally non-essential genes. For example, the virulent bacteriophage, T7, has several genes that encode functional proteins (ligase, protein kinase, host restriction blocking protein) that can be artificially deleted while maintaining T7 viability on laboratory host strains [59].

The same is true of the lysogenic bacteriophage λ [60]. However, progressive deletion of these genes causes a progressive loss of DNA packaging efficiency for λ and presumably other bacteriophages with unique DNA ends, because of a "partially full capsid" requirement for DNA packaging [60]. A result is a gene-retaining selective pressure that is independent of what the genes encode. This pressure is used in the design of bacteriophage-based gene cloning vectors (review [60]). Other mechanisms for the non-gene-specific retention of genes may exist. Possibilities include the embedding of promoters in DNA packaging recognition sites, a phenomenon that is already known for T3 and T7 [33-35] (Legend to Figure 1).

Although the two-tiered selection of the prokaryotic virus complexity hypothesis is a new concept for prokaryotic evolution, two-tiered selection is not a concept that contradicts the fundamentals of previous thinking about evolution. All events proposed in the prokaryotic virus complexity hypothesis are based on random mutation and selection. No external guidance is proposed. Similarly, production of antibodies is also two tiered, in that the first tier selection produces antibodies in an immune system that itself is the product of second tier selection [61]. The second tier genes of the prokaryotic virus complexity hypothesis have been selected to reduce the randomness of evolution via retention of the first tier. A complete, mathematical description (statistical mechanics with an extended treatment of time?) might introduce some determinism into analysis of evolution. But, at this point, the theory and data are not sufficient to say how much determinism would be introduced.

The departure from past thought is illustrated by comparing cellular gene homologs to known conditionally non-essential genes, including the non-essential bacteriophage genes described above. Conditionally non-essential genes are non-essential only in the short term. They are essential in the long term because of fluctuations in either the external environment or the interior of the host cell. In the case of both bacteria and also higher organisms, numerous documented examples exist of genetically programmed adaptation to environmental fluctuations. These include adaptations to (a) utilize thermal fluctuations to obtain variable outcome, such as a variable lysogenic response, (b) introduce environmentally modulated morphogenesis, (c) introduce environmentally-stimulated increase in mutation rate and (d) introduce cyclic changes in genome organization, such as those responsible for phase variation in bacteria (review [62]). Importantly, these previously studied adaptations to environmental fluctuations occur via genes that encode systems perfected by extensive mutation and selection in the past [62]. In the case of the cellular gene homolog evolution proposed here, the same is true of the second tier genes that encode components of the biochemical systems that maintain genes that evolve in the first tier. But, in contrast to what occurs in the case of previously described adaptation to fluctuations in the environment, the first tier homolog mutation and selection occurs *de novo*, i.e., without information from past selections.

When viewed from the perspective of genomics, the prokaryotic virus complexity hypothesis is feasible based on the following evidence of DNA exchange: (a) ancient and ongoing bacteriophage origin of initially high AT bacterial genes called ORFans, including genes for some stress-induced proteins and primosome assembly proteins [63], (b) bacteriophage origin of bacterial gene islands, defined by known sequence characteristics (including dinucleotide bias), but also containing novel genes in comparatively high concentration [64] and (c) bacterial origin of bacteriophage genes (called morons) that arrive by non-homologous recombination in a context foreign by both base composition and gene expression-controlling elements [54,65].

### Testing

Studies of evolution are plagued by both absence of direct observations and presence of primarily indirect observations of nonliving fossils. In the case of bacteriophages, however, the potential exists for genome sequencing and homology-based informatic analysis of the equivalent of living fossils, i.e., comparatively un-evolved viruses. Isolation of bacteriophages in this class, including the long-genome versions, has only just begun. Almost by definition, comparatively un-evolved bacteriophages will not compete well in most circumstances. The expectation is that these bacteriophages will be found in niches (probably not in water; more likely in soil; see [66,67] for examples) that are either isolated from or hostile to the more evolved and competitive bacteriophages.

The potential also exists for further analysis by experimentally (a) determining via gene deletion the extent to which the cellular gene homologs assist the growth of long-genome bacteriophages, (b) determining via controlled evolution the external conditions (presence or absence of a microbial community, for example) in which the cellular gene homologs are retained, (c) determining via gene deletion and mutation, followed by controlled evolution, which (second tier) genes are needed to retain the cellular gene homologs and (d) measuring via controlled evolution the extent to which the cellular gene homologs evolve, if they are retained. If, for example, the cellular gene homologs provide advantage only when a virus is within a microbial community, then the cellular gene homologs should eventually be lost during propagation in a single host that is not interacting with other microbes. Experiments of this type differ from previous experiments [68-70] in which controlled evolution was performed in the absence of any aspect of a microbial community and also without any focus on the cellular gene homologs. Also, experiments of this type should be performed with newly isolated bacteriophages (certainly not T4) that have not already evolved during propagation in the laboratory.

Informatic analysis of the DNA sequence of bacteriophage living fossils (if they are found) is also expected to be productive based on the following characteristics of bacteriophages: large number, small genome and gene diversity. These characteristics have been previously reviewed [52,53,71]. The strategy is to (a) trace via sequence similarity the past history of homologous viral genes (see, for example, [50]), (b) integrate this knowledge with knowledge of the biochemistry and (c) integrate the virus sequence similarity-based gene trees with those of prokaryotes and, eventually with at least the organelle-associated genomes [72,73] of eukaryotes. Eventually, the trees will become unambiguous and detailed enough to trace the sequence of gene evolution, though evolutionary time will remain to be specified. Comparatively un-evolved viruses potentially will be useful for the analysis of pre-split [74], as well as post-split, evolution.

## 4. Implications of the hypothesis

The prokaryotic virus complexity hypothesis extends the more general concept of reticulate evolution, i.e., evolution with hybridization among different species (reticulate evolution is reviewed in [39]). Reticulation has been proposed to explain the origin of eukaryotes [75]. The possibility exists that reticulation subsequently occurred from prokaryotes to eukaryotes (see [76], for example) and that both eukaryotic virus cellular gene homologs and some (not all) of the "junk" DNA eukaryotes [77-80] have a function similar to that of the cellular gene homologs of long-genome bacteriophages. Thus, if accurate and extendable to eukaryotes, the prokaryotic virus complexity hypothesis will also explain the function of at least some eukaryotic junk DNA.

The two-tiered aspect of the hypothesis is a new concept, but is related to the concept of hierarchical evolution that has previously been applied to eukaryotes and their communities [81]. This aspect of the hypothesis is a foundation for producing evolutionary leaps in complexity and, if found to be accurate, would be an explanation of the phenomenon of punctuated equilibrium (review [21]).

In the case of prokaryotes and their communities, the prokaryotic virus complexity hypothesis provides an intellectual framework for both understanding and influencing evolution. For example, desired changes in microbial communities might be introduced via long-genome viruses, rather than via microbial cells.

## Competing interests

The author(s) declare that they have no competing interests.

## Authors' contributions

Both the ideas presented here and articulation of these ideas are the product of the author's work.
